# Equidade entre Sexos no Acesso à Reperfusão no Infarto Agudo do Miocárdio: Um Longo Caminho a ser Percorrido

**DOI:** 10.36660/abc.20210082

**Published:** 2021-04-08

**Affiliations:** 1 Universidade Federal de Minas Gerais Faculdade de Medicina Belo HorizonteMG Brasil Faculdade de Medicina, Universidade Federal de Minas Gerais, Belo Horizonte, MG - Brasil.; 2 Universidade Federal de Minas Gerais Hospital das Clínicas Belo HorizonteMG Brasil Hospital das Clínicas, Universidade Federal de Minas Gerais, Belo Horizonte, MG - Brasil.

**Keywords:** Isquemia Miocárdica, Infarto do Miocárdio, Revascularização Miocárdica, Reperfusão Miocárdica, Mulheres, Homens

A doença isquêmica do miocárdio é a principal causa de morte no mundo. Sua apresentação de maior gravidade é o infarto agudo do miocárdio com supradesnivelamento do segmento ST (IAMCSST), que corresponde a cerca de 1/3 das apresentações^1^ e tem na reperfusão precoce a principal estratégia para a redução da mortalidade. Apesar do amplo conhecimento histórico das diferenças relacionadas ao sexo no tratamento e prognóstico de pacientes que apresentam doença cardíaca isquêmica em caráter agudo, os homens, além de terem acesso mais precoce aos sistemas de saúde, são mais propensos a receber angiografia coronária diagnóstica e revascularização de urgência do que as mulheres.^2^^-^^4^ O artigo “Acesso às terapias de reperfusão e mortalidade em mulheres com infarto agudo do miocárdio com supradesnivelamento do segmento St: Registro VICTIM^5^” mostra detalhadamente esta diferença na realidade brasileira.

Ao avaliar 878 pacientes admitidos com IAMCSST em Sergipe, estado do Nordeste brasileiro, constatou-se que as mulheres foram menos frequentemente submetidas às estratégias de reperfusão quando comparadas aos homens, tanto em intervenções coronarianas percutâneas (ICP) de urgência (44% x 54,5%; p = 0,003) quanto em fibrinólise (1,7% x 2,6%; p = 0,422). Tal realidade está em consonância com os dados de outros estudos prévios realizados sobre o tema em diferentes cenários (Tabela 1). No registro VICTIM,^5^ foi observada maior mortalidade hospitalar em pacientes do sexo feminino (16,1% x 6,7%; p < 0,001), provavelmente como consequência deste menor acesso a terapias de reperfusão. Tais dados são concordantes com revisões sistemáticas da literatura sobre o tema.^6^

**Tabela 1:** Acesso às terapias de reperfusão no infarto agudo do miocárdio com supradesnivelamento do segmento ST, por sexo

Estudo	Registro VICTIM^5^	Radovanovic et al.^11^	Hansen et al.^12^	Soeiro et al.^1^
ICP (%, homens x mulheres)	54,5 x 44	36,6 x 27,2	58 x 72	44,9 x 35,4
Fibrinólise (%, homens x mulheres)	2,6 x 1,7	18,7 x 15,2	N/A	N/A
Mortalidade (%, homens x mulheres)	16,1 x 6,7	10,7 x 6,3	11 x 7	3,7 x 3,1

ICP: intervenção coronariana percutânea.

Seria a demora pelo pedido de socorro um dos motivos pelos quais as mulheres seriam menos submetidas às terapias de revascularização? O estudo em questão demonstrou que este parece não ter sido o problema em Sergipe, sendo o tempo do chamado por ajuda após início dos sintomas estatisticamente similar entre os gêneros. No entanto, as pacientes do sexo feminino tiveram maior atraso no percurso entre o hospital primário e a remoção para uma unidade com infraestrutura para a reperfusão percutânea (atraso de transferência). Tais dados diferem, em parte, dos achados em recente estudo na Itália. No país europeu, o tempo médio do início dos sintomas até a apresentação no hospital foi maior para mulheres (280 x 240 minutos), em que apenas 23,2% das mulheres x 29,1% dos homens apresentavam atraso menor que 120 minutos até a admissão (p = 0,002).^7^ Assim como no estudo VICTIM, houve impacto sobre a mortalidade: nos casos com atraso igual ou maior que 120 minutos, as taxas de mortalidade foram maiores entre as mulheres (5,5% x 2,8%), ao passo nos casos com apresentação menor que 120 minutos, a mortalidade foi consideravelmente mais baixa e estatisticamente similar entre sexos (mulheres 2,0% x homens 1,6%).^7^

Além disso, o presente artigo observou que o IAMCSST tende a acometer mulheres com idade mais avançada (> 63 anos) e com maior número de fatores de risco em comparação aos homens.^5^ Como demonstrado em publicação prévia com dados do Brasil, as mulheres também apresentam um maior nível de estresse, o que pode potencializar o risco de eventos agudos.^8^ Padrão semelhante foi observado em estudos na Austrália e Itália, eles mostraram que as mulheres são mais propícias a apresentarem IAMCSST com idade superior e maior prevalência de hipertensão, diabetes e/ou hipercolesterolemia, o que contrasta com menores taxas de tabagismo.^7^^,^^9^ Nestas publicações questiona-se se a subestimação do risco cardiovascular feminino pode ter resultado em tratamentos mais conservadores, contribuindo para desfechos negativos.^7^^,^^9^ De modo semelhante, outros autores interrogam se a própria idade avançada das pacientes, associada ao maior número de comorbidades e ao mesmo tempo a apresentações clínicas menos típicas, não estariam influenciando a opção pelo tratamento conservador em pacientes do sexo feminino.^10^^,^^11^

Neste contexto, os dados do estudo VICTIM também chamam atenção para a importância de um menor limiar de suspeição ao solicitar exames e indicar procedimentos invasivos para pacientes do sexo feminino.^5^ Assim como demonstrado em estudo suíço com 51.725 pacientes durante um período de 19 anos (1997-2016), a mortalidade intra-hospitalar diminuiu significativamente de 9,8% para 5,5% em homens e de 18,3% para 6,9% nas mulheres como resultado do aumento da indicação de terapias de reperfusão (trombólise ou ICP) no IAMCSST: de 60% para 93% (p < 0,001) em homens e de 45% para 90% (p < 0,001) em mulheres^12^^,^^13^ – tendo aumentado proporcionalmente mais entre homens. Tais dados reforçam a importância de um julgamento clínico detalhado e individualizado na emergência médica.

Dito isso, as implicações do estudo para a comunidade médica e o médico assistente são diretas: as múltiplas comorbidades, a idade mais avançada e as apresentações atípicas não devem ser impeditivas para a indicação de terapias de reperfusão, a análise do quadro clínico como um todo se faz necessária. Ademais, devem ser propostas políticas públicas que possibilitem um encaminhamento mais rápido desses pacientes para um serviço que possua serviço de angioplastia, além de programas de educação em saúde e conscientização sobre sintomas cardiovasculares, com foco nas mulheres. Com tais ações multifacetadas, o maior acesso à reperfusão pode ser a responsável por reduzir ainda mais a mortalidade causada por doenças cardiovasculares, sobretudo no sexo feminino, nos próximos anos.
