# Supplementary material for: Equidade entre Sexos no Acesso à Reperfusão no Infarto Agudo do Miocárdio: Um Longo Caminho a ser Percorrido
Source: Arq Bras Cardiol. 2021 Apr 8;116(4):704–5. [Article in Portuguese] doi: 10.36660/abc.20210082 (PMC8121415; doi:10.36660/abc.20210082)
Supplement: Translation

# Translation: Gender Equity in Access to Reperfusion in Acute Myocardial Infarction: Still A Long Way to Go

**Affiliations:** 1 Belo HorizonteMG Brazil Faculdade de Medicina, Universidade Federal de Minas Gerais, Belo Horizonte, MG - Brazil.; 2 Belo HorizonteMG Brazil Hospital das Clínicas, Universidade Federal de Minas Gerais, Belo Horizonte, MG - Brazil.

**Keywords:** Myocardial Ischemia, Myocardial Infarction, Myocardial Revascularization, Myocardial Reperfusion, Womens, Men

Ischemic heart disease is the main cause of death worldwide, and its most severe presentation ST-elevation acute myocardial infarction (STEMI), which corresponds to approximately 1/3 of the presentations,^1^ and early reperfusion is the main strategy for reducing mortality. Despite broad historical knowledge of gender-related differences in treatment and prognosis of patients with an acute presentation of ischemic heart disease, male individuals, in addition to having earlier access to health systems, are even more likely to undergo a diagnostic coronary angiography and urgent revascularization than women.^2^^-^^4^ The article “Access to Reperfusion Therapies and Mortality in Women with Acute Myocardial Infarction with ST-Segment Elevation: the VICTIM Registry^5^” demonstrates very well this difference in access to reperfusion therapies in the Brazilian scenario. When evaluating 878 patients admitted with STEMI in the northeastern state of Sergipe, Brazil, it was observed that female individuals were less frequently submitted to reperfusion strategies when compared to males, both primary percutaneous coronary interventions (PCI) (44% x 54.5%; p = 0.003) and fibrinolysis (1.7% x 2.6%; p = 0.422). This scenario is in line with data from other previous studies on this topic carried out in different settings (Table 1). In the VICTIM^5^ registry, higher in-hospital mortality was observed in the female gender (16.1% x 6.7*; p <0.001), probably as a consequence of this lower access to reperfusion therapies. These data are consistent with systematic reviews of the literature on the topic.^6^

**Table 1:** Access to reperfusion therapies in acute myocardial infarction with ST-segment elevation, by gender

Study	VICTIM Registry^5^	Radovanovic et al.^11^	Hansen et al.^12^	Soeiro et al.^1^
PCI (%, men x women)	54.5 x 44	36.6 x 27.2	58 x 72	44.9 x 35.4
Fibrinolysis (%, men x women)	2.6 x 1.7	18.7 x 15.2	N/A	N/A
Mortality (%, men x women)	16.1 x 6.7	10.7 x 6.3	11 x 7	3.7 x 3.1

PCI: percutaneous coronary intervention.

Would the delay in calling for help be one of the reasons why women are less frequently submitted to revascularization therapies? The aforementioned study demonstrated that this does not seem to have been the problem in Sergipe, with the time spent calling for help after symptom onset being statistically similar between the genders. However, female patients underwent a greater delay in the primary hospital, until referral to a unit with infrastructure to perform the percutaneous reperfusion (transfer delay). These data differ in part from the findings of a recent study carried out in Italy, in which the mean time from symptom onset to presentation at the hospital was longer for women (280 x 240 minutes), with only 23.2% of women x 29.1 % of men undergoing a delay <120 minutes until hospital admission (p = 0.002).^7^ As in the VICTIM study, there was an impact on mortality: in cases with delay ≥120 minutes, mortality rates were higher among women (5.5% x 2.8%), whereas in cases with presentation <120 minutes, mortality was considerably lower and statistically similar between genders (2.0% in women vs. 1.6% in men).^7^

Moreover, the present study observed that STEMI tends to affect women who are older (> 63 years) and with a greater number of risk factors, when compared to men.^5^ As demonstrated in a previous publication with data from Brazil, women also have a higher level of stress, which may increase the risk for acute events.^8^ A similar pattern was observed in studies carried out in Australia and Italy, which showed that women are more likely to have STEMI at an older age and to have higher rates of hypertension, diabetes and/or hypercholesterolemia, in contrast to lower rates of smoking.^7^^,^^9^ In these publications, it is questioned whether the underestimation of female cardiovascular risk may have resulted in more conservative treatments, contributing to unfavorable outcomes.^7^^,^^9^ Similarly, other authors question whether the patients’ advanced age, associated with a greater number of comorbidities, together with less typical clinical presentations, would influence the choice of conservative treatment in women.^10^^,^^11^

In this context, the data from the VICTIM study also draw attention to the importance of a lower threshold of suspicion for ordering tests and indicating invasive procedures for female patients.^5^ As demonstrated in a Swiss study with 51,725 patients, over a period of 19 years (1997 - 2016), in-hospital mortality significantly decreased from 9.8% to 5.5% in men and from 18.3% to 6.9% in women, as a result of the increasing indication for reperfusion therapies (thrombolysis or PCI) in STEMI: from 60% to 93% (p <0.001) in men and from 45% to 90% (p <0.001) in women^12^^,^^13^ – with a proportionally greater increase among men. These data reinforce the importance of a detailed and individualized clinical judgment in the emergency setting.

That said, the study implications for the medical community and attending physicians are straightforward: the multiple comorbidities, older age and atypical presentations should not be a barrier to the indication of reperfusion therapies, and the clinical condition should be analyzed as a whole. Additionally, public policies should be proposed to allow for a faster referral of these patients to a service with intervention a facilities, in addition to health education programs and awareness of cardiovascular symptoms, focused on women. With these multifaceted actions, greater access to reperfusion may result in further reductions in mortality from cardiovascular diseases, especially among women, in the coming years.
